# Polyanilines as New Sorbents for Hydrocarbons Removal from Aqueous Solutions

**DOI:** 10.3390/ma13092161

**Published:** 2020-05-07

**Authors:** Cristina Della Pina, Maria Antonietta De Gregorio, Pierluisa Dellavedova, Ermelinda Falletta

**Affiliations:** 1 Dipartimento di Chimica, Università degli Studi di Milano, via C. Golgi, 19, 20133 Milano, Italy; cristina.dellapina@unimi.it; 2 Settore Laboratori, ARPA Lombardia, via Rosellini, 17, 20124 Milano, Italy; a.degregorio@arpalombardia.it (M.A.D.G.); p.dellavedova@arpalombardia.it (P.D.)

**Keywords:** water remediation, porous polyaniline, hydrocarbons, green synthesis

## Abstract

Water remediation from hydrocarbons is crucial to reduce health risks. Numerous costly and, sometimes, sophisticated methods were proposed over the years. Herein, an innovative green procedure for porous polyanilines preparation is reported. Polyaniline (PANI) was synthesized by three different approaches ranging from traditional to more eco-friendly ones. Thermal, optical and morphological features of the resulting materials were investigated along with their surface properties. Finally, PANIs were tested as sorbents for hydrocarbons removal from waterbodies. Although an overall fast and high sorption efficiency is always observed, the effective hydrocarbons abatement performed by ‘green’ PANIs is particularly welcome in the context of environmental protection. Moreover, the sorption efficiency retention after five-run recycling tests suggests potential applications in wastewater remediation.

## 1. Introduction

**Figure 1:**
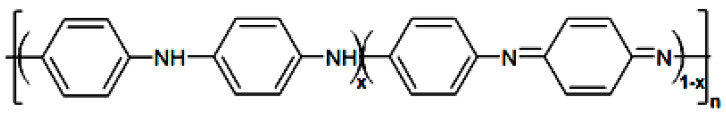
Molecular structure of polyaniline (PANI).

In the context of environmental defense, wastewater remediation from organic pollutants represents a major objective stimulating the material sciences. An efficient sorbent material should display specific textural and morphological properties to promote the absorption process, such as large surface area and porosity. For this reason, some materials such as activated carbons [1], polymeric membranes [2] and metal oxides [3] are generally included in the selected shortlist, although they suffer from some limitations. To give some examples, reactor pressure drop, high regeneration temperature and difficult recovery after use. Among the innovative materials proposed to replace traditional activated carbons, polyaniline (PANI, Figure 1) plays an important role owing to its peculiar features, as facile synthesis, strong stability and marked reduction-oxidation properties [4,5,6].

Polyaniline is traditionally synthesized starting from the monomer (aniline) by oxidative polymerization with noxious stoichiometric oxidants (typically metals in high oxidation states) [7]. Although this approach leads to high quality material in terms of chemical-physical properties, toxic and cancerous benzidine and *trans*-azobenzene are achieved in large amounts as co-products, as well as inorganic waste (sulphates, chromates, heavy metals, etc.). As a consequence, extensive purification steps are required. For this reason, we have recently proposed more environmentally friendly alternatives whose synthetic routes follow the oxidative polymerization of aniline dimer (*N*-(4-aminophenyl)-aniline). Oxidants such as the eco-benign hydrogen peroxide or molecular oxygen were used with the aid of selected catalysts [8,9,10,11,12]. These novel approaches lead only to H_2_O as the co-product thus avoiding noxious species release and streamlining post-treatment.

In addition to the ability of PANI to remove aromatic hydrocarbons (e.g., dyes) [8,9,10,11,12,13] and metals (e.g., heavy metals) [14], we recently demonstrated that it can be also employed for VOCs (Volatile Organic Compounds) sampling or abatement/adsorption purposes [6]. Herein, we present an innovative environmentally friendly one-step procedure for porous PANIs preparation. Furthermore, we introduce our recent results in hydrocarbons (HC) removal from aqueous solutions using three different types of PANI. To the best of our knowledge, this is the first study in which polyaniline is employed as sorbent material for water remediation from HC. Dodecane (C12), a model molecule for HC, was selected as a target pollutant to investigate the sorption activity of different PANIs. The synthesized materials were then applied in mineral oil (MO) removal. Considering that a HC leakage causes water pollution quickly and in a broad-ranging area, removal kinetics was investigated as well as recycling tests were carried out demonstrating fast sorption ability and high efficiency retention.

## 2. Materials and Methods

All chemicals were bought by Merck and did not undergo purification processes. A certified reference material (CRM) of mineral oil (MO, standard solution of 8 mg/mL in heptane (O2Si)) was used both for sorption evaluation and GC calibration.

### 2.1. Materials Preparation

#### 2.1.1. PANI1 Synthesis

Following the procedure reported in the literature [7], 10 mL of anilinium hydrochloride solution was prepared adding 1.0 g of aniline in 10 mL of HCl 10 M. The solution was cooled at 4 °C. After 30 min, 15 mL of an aqueous solution of (NH_4_)_2_S_2_O_8_ 1.29 M was dropwise added to the anilinium hydrochloride solution. The mixture was stirred for 6 h. Then, the reaction was stopped by the addition of acetone. A solid material was filtered, washed several times by water and acetone and dried under air.

#### 2.1.2. PANI2 Synthesis

According to a previous synthetic procedure reported elsewhere [11], PANI2 was prepared as follows. *N*-(4-aminophenyl)-aniline (2 g) was weighted and added in 200 mL of 0.50 M HCl at room temperature. The oxidative polymerization reaction took place after the quick addition of 5.6 mL of H_2_O_2_ 30%, followed by 0.1 mL of a solution of Fe^+3^ 10 mg/mL (1.0 m). The mixture was stirred for 24 h. Finally, a solid material was collected and treated as described in Section 2.1.1.

#### 2.1.3. PANI3 Synthesis

N-(4-aminophenyl)-aniline (2 g) was dissolved in 200 mL of a mixture EtOH/H_2_O (30/70 *v*/*v*). Then, 4.5 mL of HCl 37% was added, followed by 5.6 mL of H_2_O_2_ 30% and 0.1 mL of a solution of Fe^+3^ 10 mg/mL (1.0 mg). The reaction was stirred for 24 h at room temperature. Then, a solid material was collected and treated as described in Section 2.1.1.

### 2.2. Materials Characterization

Fourier Transform Infrared (FT-IR) spectra were collected using a JASCO FT/IR-410 spectrophotometer (JASCO Corporation, Tokyo, Japan) in the transmittance mode. A small amount of sample was mixed with KBr and finely crushed. The resulting powder was compressed in 13 mm diameter pellets with a 10-ton hydrostatic press for 10 min. The obtained pellets were analyzed in the 400–4000 cm^−1^ range with 20 scans at a resolution of 4 cm^−1^.

A Hewlett Packard 8453 spectrophotometer (Hewlett Packard Gmbh, Waldbronn, Germany) was employed to record UV-vis absorption spectra in the 200–1000 nm range with a resolution of 1 nm. Each sample was dissolved in DMF (dimethylformamide) in 1 cm path length quartz cuvettes

For the X-ray powder diffraction (XRPD) analyses a Philips PW 3710 Bragg-Brentano goniometer (Philips, Amsterdam, Netherlands) equipped with a scintillation counter, a slit with 1° divergence, a receiving slit of 0.2 mm and a 0.04° Soller slit system. A graphite-monochromatic Cu Kα radiation was adopted at a nominal X-ray power of 40 kV × 40 mA. Diffractograms were obtained in a 2θ range between 10° and 80°.

The Brunauer–Emmett–Teller (BET) method was adopted to determine the composite specific surface area from adsorption isotherms of N_2_ in subcritical conditions, measured using a Coulter SA3100 instrument (Beckman-Coulter, Brea, CA, USA). The pore size distribution of selected samples was determined via the Barrett–Joyner–Halenda (BJH) method from the desorption isotherms. Before measuring, each sample was outgassed at 150 °C for 2 h under vacuum.

A Zeiss LEO 1430 instrument (Zeiss, Jena, Germany) was employed to acquire Scanning Electron Microscopy (SEM) images.

Thermogravimetric analyses (TGA) were performed in air (5 °C/min, temperature range 30–900 °C) with a TGA/DSC 3+ Mettler Toledo instrument (Mettler Toledo, Greifensee, Switzerland) equipped with a 70 μL alumina crucible.

Size exclusion chromatography (SEC) technique was employed to estimate the molecular weight distributions of PANIs extracts by dimethylformamide (DMF). Sample for SEC analysis was prepared dissolving approximately 10 mg of the material in 5 mL of DMF under sonication for 20 min at room temperature. Only the soluble fraction was analyzed, after filtration. Of each extract, 20 µL was injected in a Shimadzu LC10ADVP HPLC (Shimadzu Corp., Ltd., Kyoto, Japan) equipped with a refractive index (RI) as the detector. The chromatographic column was a Phenomenex Phenogel 5u 55 A (300 × 4.6 mm, Phenomenex, Torrance, CA, USA) and operated at room temperature. Ultra-pure DMF was used as the eluent and the flow rate was maintained at 0.3 mL/min. Calibration was carried out using polystyrene standards.

GC/FID analyses were performed on 6890A–LTM–PTV–FID Agilent (Agilent Technologies Inc., Wilmington, DE, USA) equipped with a LTM column (J&W DB-5 5 m, 0.32 mm, 1.0 µm).

GC/FID conditions are reported below and all the extracted fractions were quantified by the external standard method.

GC oven: 340 °C; column LTM program: 40 °C for 1 min, then 20 °C/min to 280 °C for 0 min; then 100 °C/min to 340 °C for 2 min. Gas carrier: helium. PTV inlet: Mode Pulsed Splitless. Inlet temperature: 320 °C. Pulsed pressure: 30 psi for 0.2 min; Purge Flow to split Vent: 100 mL/min a 0.5 min. Ramp pressure: 17.4 psi for 0.5 min, then 5.8 psi/min at 23.2 psi for 0 min, then 2.9 psi/min at 26.1 psi for 0.5 min. Temperature detector (FID): 340 °C, H_2_ flow: 40.0 mL/min, air flow: 400.0 mL/min, makeup flow: 25.0 mL/min.

### 2.3. Sorption Experiments

An amount of 1 mg of each PANI was dispersed in 100 mL of water contaminated with *n*-dodecane (0.75 mg and 75 mg) or mineral oil (0.8 mg) under stirring for 5, 20 and 60 min. Then, the polymer was recovered by centrifugation and the water/oil emulsion was extracted three times with about 5 mL of heptane. The organic extracts were reunited, diluted at 25 mL and analyzed by GC/FID.

Each PANI (1 mg) was treated as described above but in the absence of any hydrocarbon to evaluate blank level.

### 2.4. Recycling Tests

PANI2 and PANI3, after being tested as reported in Section 2.3, were subjected to 5 consecutive sorption tests without any regeneration process.

## 3. Results and Discussion

### 3.1. Characterization of PANIs

In order to get insights into the effect of the reaction conditions on the polymers structure and morphology, FT-IR, UV-vis and TGA analyses were carried out along with surface area and SEM investigations.

**Figure 2:**
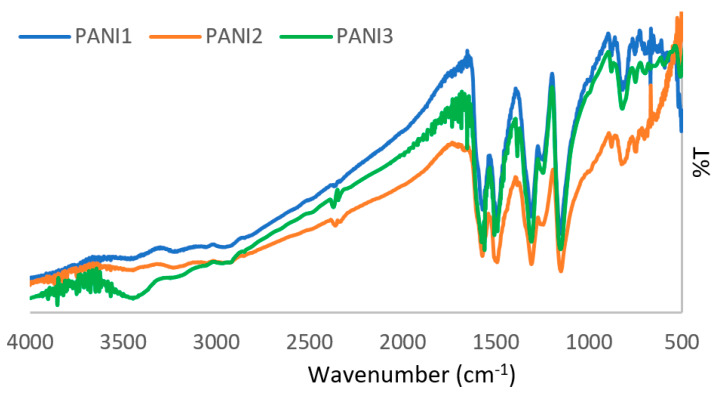
FT-IR spectra of PANI1, PANI2 and PANI3.

Figure 2 reports the FT-IR spectra of all the materials.

FT-IR spectra of all the polymers show the characteristic bands of PANI in its conductive emeraldine form. Briefly, C=C stretching of the quinoid rings (N=Q=N) is responsible for the band at 1571 cm^−1^, whereas the C=C stretching vibration modes of the benzenoid rings (N-B-B) causes the two bands at 1508 cm^−1^ and 1490 cm^−1^. The bands at 1309 cm^−1^ and 1245 cm^−1^ can be attributed to the C-N and C=N stretching modes. The in-plane and out-of-plane bending of C-N is responsible for the bands at 1036 cm^−1^ and 881 cm^−1^. Finally, the deformation vibration modes for the aromatic rings are related to the bands at 750 cm^−1^ and 691 cm^−1^, whereas that at 573 cm^−1^ can be assigned to 1,4 di-substituted benzenes [11].

**Figure 3:**
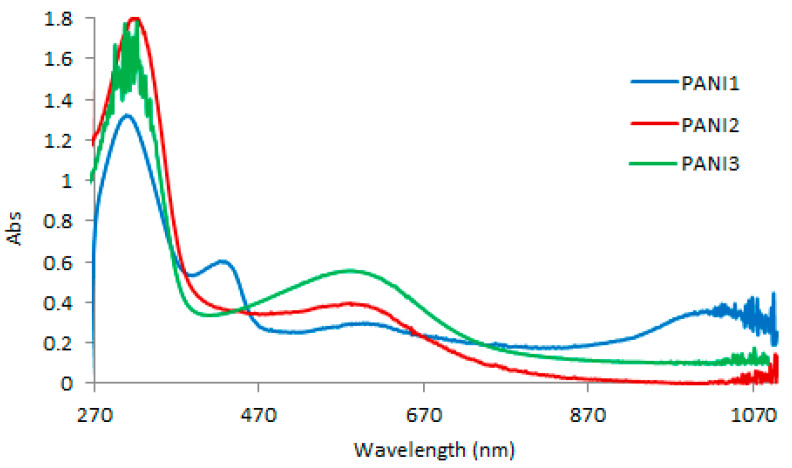
UV-vis spectra of PANI1, PANI2 and PANI3 in dimethylformamide (DMF) solution.

As it is possible to observe from Figure 3, the electronic spectra of all the synthesized materials exhibit typical absorption bands of deprotonated emeraldine: a first band at about 300 nm, related to the π→π* transition of benzenoid rings, due to the effect of a conjugation extension between adjacent aromatic rings, and a second band at about 600 nm, connected to the formation of excitons from benzenoids to quinoids, but attributed also to charge transport that can occur between neighboring chains or within the same chain [6,8].

The UV-vis spectrum of PANI1 shows two new bands. π-polaron and polaron-π* transitions, related to the presence of excitation polaron states (charged cation radicals,), are responsible for the first band at about 420 nm, while different factors could be responsible for the second band, as reported in the literature [11].

**Figure 4:**
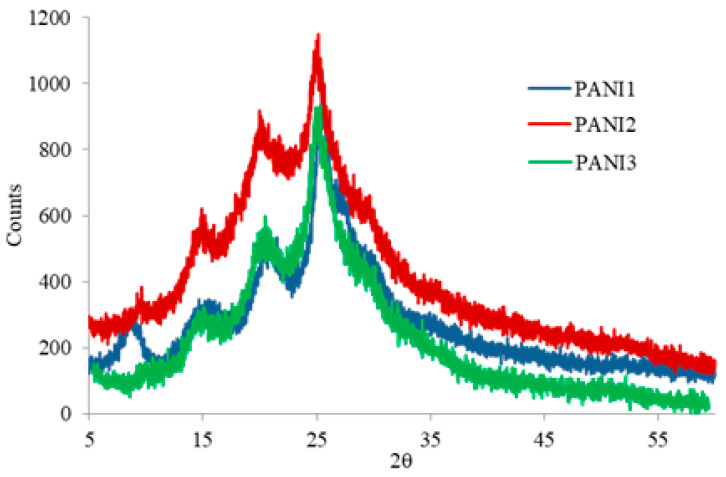
XRPD patterns of PANI1, PANI2 and PANI3.

No structural differences among the PANIs were observed by XRPD investigations (Figure 4).

The diffraction peaks of all the PANIs in Figure 4 are in line with those typical of emeraldine salt in ES-I form [15], that is the protonated PANI form obtained from solution.

In more detail, three main peaks are visible at 2θ = 14.8°, 20.3° and 25.0°, assigned to the repeat unit of the PANI chain and the periodicity perpendicular and parallel to the polymer backbone chain, respectively. The peaks correspond to (011), (020) and (200) crystallographic planes reflections of PANI in its emeraldine salt (PANI-ES).

The presence of an additional peak at 2θ~10° in the XRPD pattern of PANI1 suggests a crystalline domain increase in the polymeric matrix [15].

**Figure 5:**
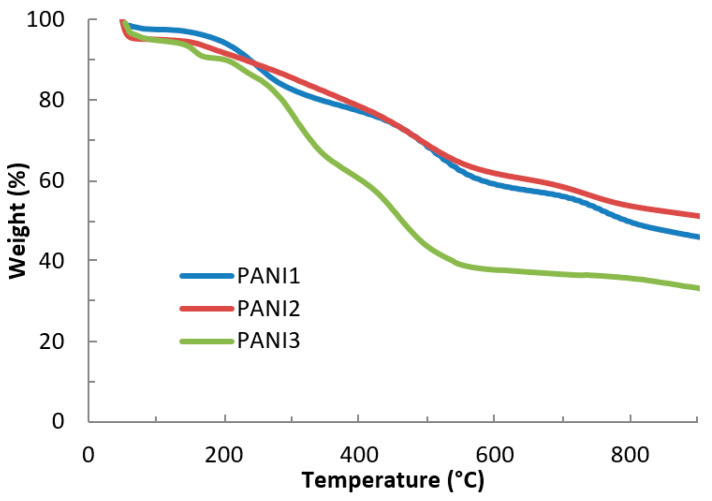
TGA curves of PANI1, PANI2 and PANI3.

On the contrary, important differences were observed by thermal degradation investigations (Figure 5).

In agreement with the literature [16], TG analyses of all the materials performed in air show four main weight loss stages: the first one at around 100 °C is caused by the loss of physisorbed water, the loss of the dopant (HCl) is responsible for the second one (150–300 °C), the third one (350–600 °C) is caused by oligomeric chains degradation and finally the fourth one, starting at around 600 °C, is attributed to the polymer backbone degradation. The results show that, if compared to PANI1 and PANI2, exhibiting a very similar thermal behavior, the thermal degradation profile of PANI3 is characterized by important losses between 300 and 600 °C. However, all the investigated materials show an interesting thermal stability.

The thermal behavior of PANI can be ascribed to numerous different factors, such as structure of the polymer backbone, oxidation state, length of the chains, morphology, etc. [16,17,18,19].

Concerning polymeric structure, no differences were detected among the three materials, as demonstrated by the spectroscopic investigations reported in Figure 2. Only the higher intensity of the UV-vis band at about 600 nm in the PANI3 spectrum, related to the presence of quinoid rings, could be correlated to the lower thermal stability of this polymer. PANI-based materials exhibit poor solubility in ordinary organic solvents, the investigation of their molecular weight is problematic and controversial. Here, we report the molecular weights of the PANIs extracts by DMF estimated via SEC, using polystyrene standards. It is worth noting that hydrodynamic volumes of polystyrene and PANI are different [20]. Therefore, the results represent apparent molecular weights and not absolute ones. However, the results confirm that all the synthesized PANIs had high and similar values of molecular weight (1.7 × 10^5^ Da, 3.0 × 10^5^ Da and 2.7 × 10^5^ Da for PANI1, PANI2 and PANI3, respectively).

**Figure 6:**
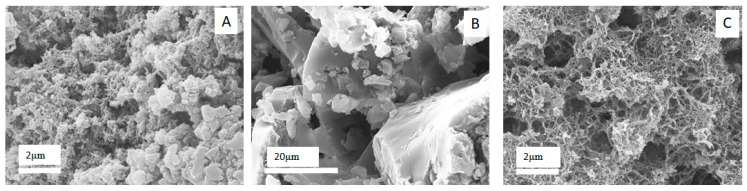
SEM images of PANI1_HCl (**A**), PANI2_HCl (**B**), PANI3_HCl (**C**).

Surprisingly, regarding PANIs morphology, extraordinary differences were observed among the materials by SEM investigations. Figure 6 shows that both PANI1 and PANI3 are characterized by a porous nanorod-based morphology, whereas PANI2 exhibits a smooth and compact morphology.

Although the mechanism of PANI synthesis is still not completely clear, it can be considered as a two-step reaction, where the first step is responsible for the formation of active radical species (monomers and dimers), while during the second step chain elongations occur. The present results show that the capability to control the first reaction step is crucial to produce well organized structures. In fact, as reported in the literature [21], a fast radical reaction is responsible for the oxidative polymerization of aniline monomer (PANI1). Therefore, if this step is not strictly controlled, side-reactions could take place negatively affecting PANI quality (e.g., morphology, conductivity and porosity), because of branching and/or overoxidizing chain reactions. However, the first step of this reaction (oligomers formation) is slow enough to permit the reaction control and polymer growth. When *N*-(4-aminophenyl)-aniline is used as the reagent (PANI2), the first reaction step speeds up, making any reaction control hard and leading to an unorganized polymeric elongation with negative consequences on the morphology. In this regard, the addition of a radical scavenger in the proper amount, such as an alcohol, allows a more precise control over the radical formation, thus reducing their uncontrolled proliferation and permitting more regular chains growth (PANI3), although this is detrimental for the yield. In fact, if on the one hand PANI1 and PANI2 were obtained in high yield (PANI1 92%, PANI2 80%), on the other hand the yield of PANI3 synthesis was lower (ca. 60%).

Finally, specific surface areas measurement confirmed the SEM results, displaying for PANI1 a value of 32.1 m^2^/g, for PANI2 2.5 m^2^/g and 50.0 m^2^/g for PANI3.

On the basis of these results and in agreement with our previous observations [22], it is possible to conclude that the structural characteristics of these materials, as well as their thermal stability, are strongly affected by the synthetic method that plays a key role in the polymers morphology.

### 3.2. HC Removal by PANIs

The sorption capability of all the synthesized materials was initially tested towards the removal of *n*-dodecane (C12) in aqueous solution and then on mineral oil (MO) abatement.

In the first part, C12 was used as a model molecule in order to set the experimental conditions and at the same time test the removal ability of the polymers towards high HC levels. In fact, in a first set of experiments 1mg of each kind of PANI was put in contact with 0.75 mg of C12 for 60 min, whereas in a second set of tests the C12 quantity was increased a hundredfold (75 mg). Because all the polymers exhibited similar activity in both cases, here only the results of the second set of experiments were reported.

For the MO abatement tests, 0.8 mg of analyte was put in contact with 1 mg of each polymer for 60 min. MO used for the experiments was a mixture consisting of equal parts of diesel fuel and lubricating oil. In both cases HC level was in line with the total petroleum hydrocarbons concentration in feeding wastewater (in the order of ppm) [23]. High values of HC/sorbent (*w*/*w*) were employed (75,000 and 800 for the abatement of C12 and MO, respectively).

Equation (1) was used to calculate sorption efficiency:

Sorption efficiency% = 100 − (conc HC not sorbed/initial conc HC × 100)(1)

**Figure 7:**
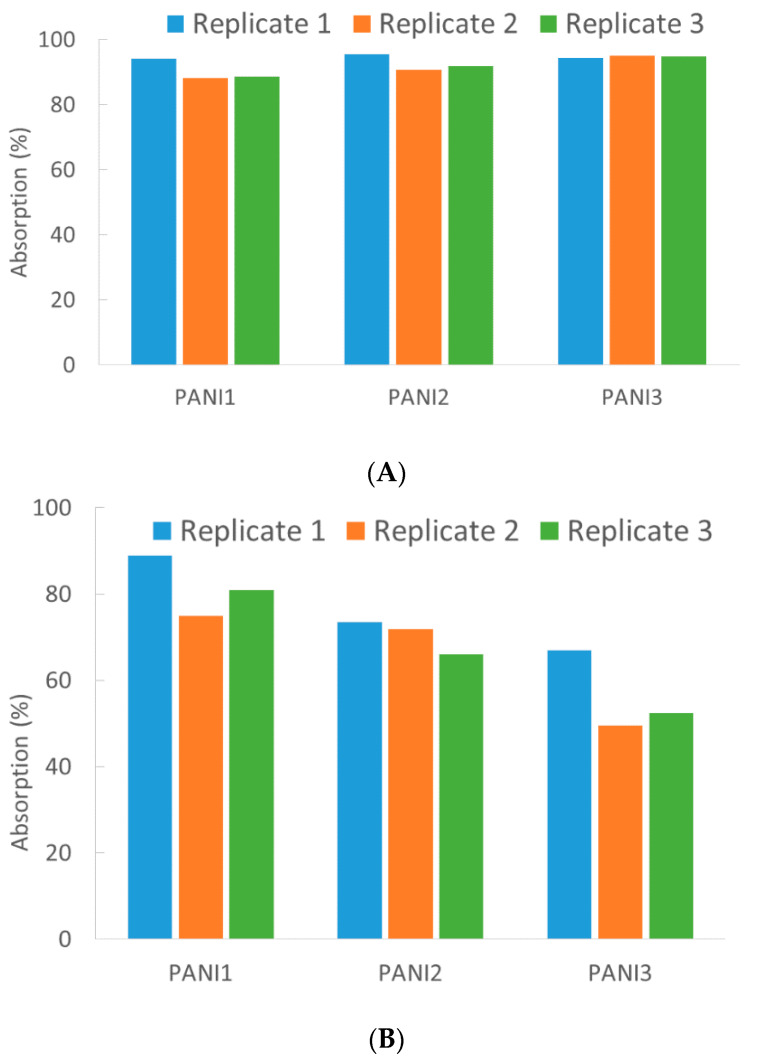
Percentage of sorption efficiency (three replicates for each material) toward *n*-dodecane (C12) (**A**) and MO (**B**) for PANI1, PANI2 and PANI3.

As displayed in Figure 7A, all the investigated PANIs show extraordinary high sorption properties towards C12 removal. In fact, HC was reduced between 88% and 94% and good reproducibility and repeatability were observed.

The sorption capacity of the three polymers was 67,500 mg/g, 69,750 mg/g and 71,250 mg/g for PANI1, PANI2 and PANI3, respectively.

In order to evaluate the minimum contact time necessary to reach interesting sorption activity, the experiments were repeated by reducing the contact time.

For all the three materials, the highest sorption efficiency was reached in a few minutes. Unexpectedly, the most porous materials (PANI1 and PANI3) diplayed the maximum sorption efficiency (94%) after 20 min, whereas the most compact polymer (PANI2) was already active after 5 min (95% sorption). Longer contact times did not cause higher uptakes. The different sorption ability of the three materials is more evident in the MO removal (Figure 7B).

As reported in the literature [24,25], different mechanisms can be involved in oil sorption, such as adsorption, absorption, or both. In addition to these, other interactions may take part in the uptake, for example cohesion and adhesion phenomena.

On the basis of the results showing similar behavior both for porous (PANI1 and PANI3) and compact (PANI2) materials and considering the low surface areas, especially for PANI2, it is possible to hypothesize that all the previously reported mechanisms are involved in oil removal. If for the most porous polymers (PANI1 and PANI3) adsorption and absorption mechanisms could be prevalent, cohesion/adhesion mechanisms would prevail in PANI2–HC interactions leading to oil layers on the polymer.

Accordingly, even though PANI1 displays higher sorption efficiency, PANI2 results in the best reproducibility and repeatability, whereas PANI3 shows poorer sorption capability as well as low reproducibility and repeatability. Such results turn out to be of high interest if compared with similar experiments reported in the literature using different materials [26,27]. For MO abatement the polymers showed sorption capability values of 654 mg/g (PANI1), 563 mg/g (PANI2) and 450 mg/g (PANI3). A strict comparison between the presented results and the data reported in the literature is not easy due to different chemical-physical properties of the materials. Neverthless, it is worth noticing that the sorption uptake of the three PANIs is extraordinarily high even for C12 and MO. Differently, the oil sorbent capacity ranges from 0.1 g/g to 5 g/g for some inorganic materials [27], while the values increase up to >100 g/g for highly porous compounds, such as foams and fibers [24]. Concerning reproducibility, an inevitable analytical variability has to be considered (numerous reference methods assumes about 30% bias variation) [28], along with a certain lack of homogeneity of the porous materials (PANI1 and PANI3).

### 3.3. Recycling Tests

**Figure 8:**
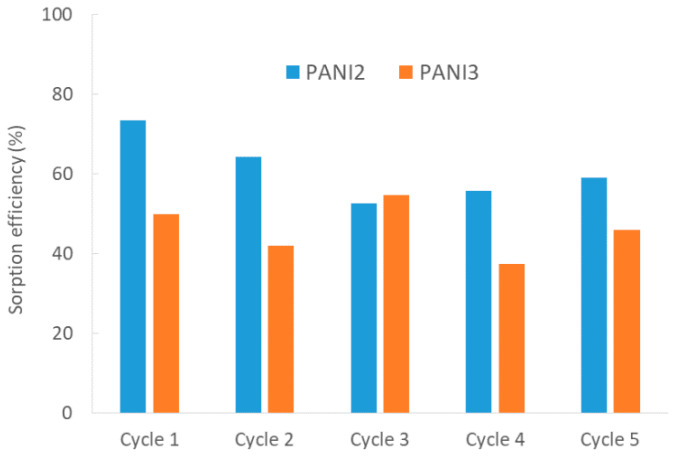
Percentage of sorption efficiency towards MOs for PANI2 and PANI3 under 5-run recycling (cycles 1–5).

In order to reduce waste production and process costs, being able to count on regenerate and reusable sorbent materials is crucial. For these reasons, on the basis of the good performances obtained for PANI2 and in order to open new perspectives for PANI3 applications, five-run recycling tests of these materials were carried out (Figure 8).

The results of Figure 8 display good reusability for both the PANIs also after five consecutive tests, guaranteeing good stability of the materials under the investigated conditions and promoting them as a good alternative to traditional sorption materials. As previously observed for the materials’ sorption efficiency (Figure 7), there is also a certain data variability raised which is ascribable to the materials’ dishomogeneity, as well as to normal analytical processes.

## 4. Conclusions

The eco-friendly preparation of an innovative porous PANI was described and compared to other synthetic routes. Three different polymeric materials were achieved and tested in hydrocarbons removal from water. All the PANIs exhibited high sorption ability, which was demonstrated herein for the first time. The sorption uptake of PANIs is halfway between inorganic materials and those characterized by low porosity and highly porous organic materials. Importantly, PANIs prepared by innovative green approaches (PANI2 and PANI3) retained good performances even after five-run recycling. These achievements can pave the way to their employment in wastewater remediation, as an alternative to traditional materials presently used.
